# ﻿Taxonomic revision of the peculiar genus *Xylopodia* (Loasaceae) with a new species from Argentina and Bolivia demonstrating an atypical trans-Andean disjunction

**DOI:** 10.3897/phytokeys.194.77827

**Published:** 2022-04-15

**Authors:** Claudia M. Martín, Christian A. Zanotti, Rafael Acuña-Castillo, Tilo Henning, Juan C. Catari, Maximilian Weigend

**Affiliations:** 1 Centro de Investigaciones y Estudios en Diversidad Vegetal (CIEDIVE), Facultad de Ciencias Agrarias, Universidad Nacional de Jujuy, Alberdi 47, 4600, Jujuy, Argentina; 2 Instituto de Ecorregiones Andinas (CONICET-UNJu), Canónigo Gorriti 237, 4600, Jujuy, Argentina; 3 Instituto de Botánica Darwinion, IBODA-CONICET, Labardén 200, Casilla de Correo 22, B1642HYD, San Isidro, Buenos Aires, Argentina; 4 Escuela de Biología, Universidad de Costa Rica, Apdo. Postal 11501–2060 San Pedro de Montes de Oca, San José, Costa Rica; 5 Herbario Luis A. Fournier Origgi, Centro de Investigación en Biodiversidad y Ecología Tropical (CIBET), Universidad de Costa Rica, Apdo. Postal 11501–2060 San Pedro de Montes de Oca, San José, Costa Rica; 6 Leibniz Centre for Agricultural Landscape Research (ZALF), Eberswalder Str. 84, 15374, Müncheberg, Germany; 7 Herbario del Oriente (USZ), Museo de Historia Natural Noel Kempff Mercado, Av. Irala 565, 2489, Santa Cruz, Bolivia; 8 Nees-Institut für Biodiversität der Pflanzen, Universität Bonn, Meckenheimer Allee 171, 53115 Bonn, Germany

**Keywords:** Andes, Argentina, Bolivia, endemism, Loasoideae, taxonomy, *Xylopodia*

## Abstract

Loasaceae subfam. Loasoideae are a nearly exclusively American plant group with a center of diversity in Peru. Numerous new taxa have been described over the past decades; one of the most striking discoveries was that of the narrowly endemic *Xylopodia* with the single species *Xylopodiaklaprothioides* in Peru, Dpto. Cajamarca in 1997. Surprisingly, field studies in the past years have resulted in the discovery of material clearly belonging to the same genus in both Bolivia and northern Argentina, approximately 1500 km SE of the next known population of *Xylopodia* in Contumazá, Peru. A closer examination shows that Argentinian and Bolivian material belongs to a single species, clearly different from *Xylopodiaklaprothioides*. We here describe *Xylopodialaurensis* and the entire genus is revised. Both species are illustrated, all aspects of their biology and ecology are portrayed and their threat status is discussed.

## ﻿Introduction

**Figure 1.**
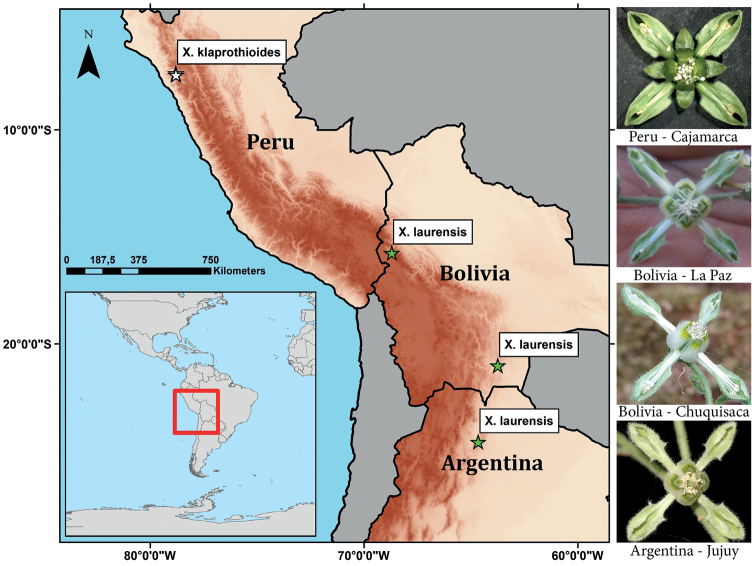
Known distribution of *Xylopodiaklaprothioides* and *X.laurensis* (photo credit for La Paz: A.F. Fuentes).

**Figure 2.**
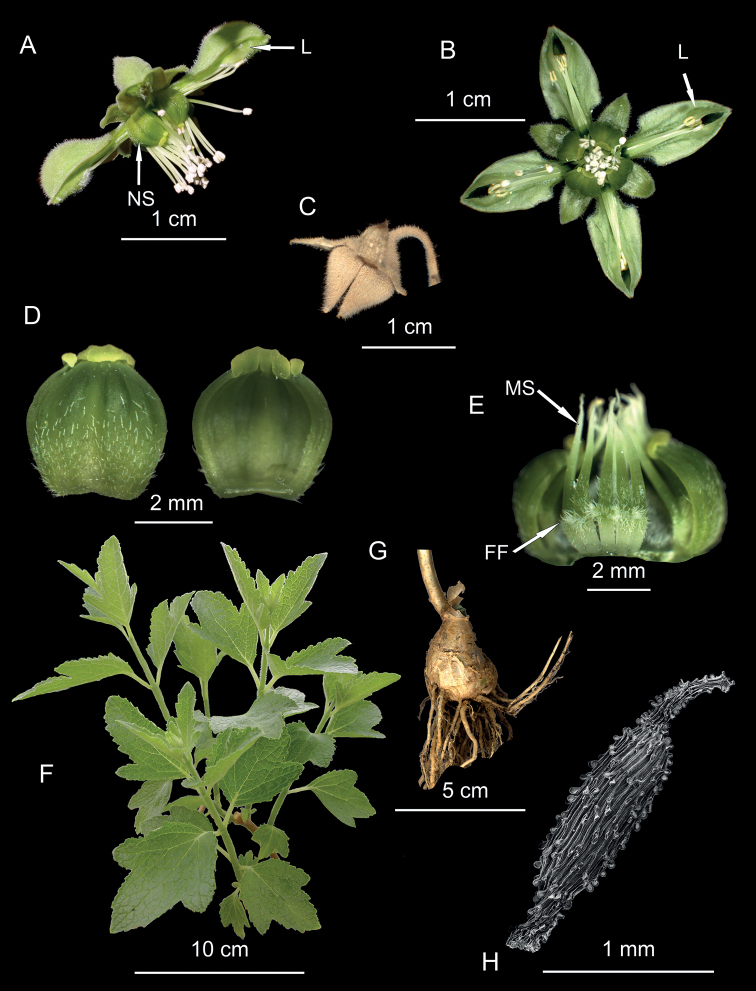
*Xylopodiaklaprothioides***A** flower, lateral view, showing the lamellae (L) and nectar scales (NS) **B** flower, frontal view, showing the lamellae (L) **C** mature fruit with slightly opened apical valves **D** nectar scale in dorsal (left) and ventral (right) views **E** nectar scale complex with one scale removed to reveal the internal monomorphic staminodes (MS) and the inconspicuous filament flanges (FF) **F** sterile branches with mature leaves **G** xylopodium of a 2-year-old plant **H** seed. All from *Weigend et al. 97/450*.

Loasaceae are a largely Neotropical family with a center of diversity in the Central Andes of Peru and neighboring Ecuador. This is particularly true for the most diverse subfamily Loasoideae (>200 spp.) and its most speciose genus, *Nasa* Weigend (>100 spp., Weigend 2004a; Henning et al. 2019). Loasaceae of southern South America are relatively well understood with several recent compilations and systematic studies available (Weigend 2007; Weigend et al. 2008; Weigend and Ackermann 2015; Acuña-Castillo et al. 2017). Most recently, a national treatment for the flora of Argentina was completed (Acuña-Castillo et al. 2021). Phylogenetic studies have largely resolved the major lineages of Loasaceae (Weigend et al. 2004; Acuña-Castillo et al. 2017; Acuña-Castillo et al. 2019) including many of the recently discovered taxa. One of the most striking discoveries of the past decades was the narrowly endemic genus *Xylopodia* Weigend, a peculiar subshrub with a thick, persistent, woody rhizome (xylopodium) and short-lived erect flowering branches. The taxon was discovered in two populations, restricted to northern Peru near the town of Contumazá (Dpto. Cajamarca, Prov. Contumazá), a region long known for its rich and diverse flora (Figs 1, 4C). Based on the characteristic morphology (especially tetramerous flowers and petals with adaxial lamellae) the plant was recognized as a close ally of widespread *Klaprothia* Kunth as member of the tribe Klaprothieae (Weigend 2006). Phylogenetic studies confirmed a close and well supported relationship between these two genera with Acuña-Castillo et al. (2019) dating the split between *Xylopodiaklaprothioides* Weigend and its sister group (*Klaprothia* + *Plakothira* J. Florence) to the mid-Oligocene (ca. 28 Ma). Although definitively part of Loasoideae, the relationships of Klaprothieae to other Loasoideae are still uncertain, although low support has been retrieved for a relationship with African *Kissenia* R. Br. ex Endl. (Acuña-Castillo et al. 2019). Since its discovery in 1997, *Xylopodiaklaprothioides* (Fig. 2) has been considered a rare paleoendemic, that survived only in a single spot on the western Andean slope in northern Peru, part of the so-called Amotape-Huancabamba-Zone, a region of extraordinary species-richness including many old relic species, disjuncts and massive recent radiations (Weigend 2002, 2004b; Weigend et al. 2005; Mutke and Weigend 2017). However, recent field studies by one of us (CM) have led to the discovery of plants that can be clearly assigned to the genus *Xylopodia* in Argentina, Prov. Jujuy, approximately 2,500 km from the closest known population in northern Peru. Also, field studies in Bolivia yielded additional sightings and collections of *Xylopodia* from both the Dptos.of La Paz (as far back as 2017 http://legacy.tropicos.org/Image/100494035) and Chuquisaca (in 2019) (Fig. 1). High-quality photographs of the living plants permitted a close comparison of the material from Peru, Argentina, and Bolivia, leading to the conclusion that the southern material can be assigned to a single species, distinct from, but similar to Peruvian *Xylopodiaklaprothioides*. Based on these findings, we describe a new species of *Xylopodia*. Its ecology, habitat, and distribution are discussed and its conservation status assessed. Lankester Composite Dissection Plates (**LCDP**) have been prepared to compare and document the most important morphological traits of both known species of this poorly-known and poorly-collected genus.

## ﻿Materials and methods

Field studies were carried out in 2019 and 2021 in Chuquisaca, Bolivia and in 2021 in Jujuy, Argentina. Photographs of the plant habit and macro-morphological characters were taken in the field with a Canon EOS 7D. Morphological observations and measurements of the new species were obtained from living and dry specimens from Argentina and Bolivia. Micro-morphological characters were observed and photographed with a Leica EZ4 stereomicroscope. Argentinian and Bolivian specimens are deposited in SI and USZ respectively. The comparison with Peruvian *X.klaprothioides* was carried out using photographs and specimens from Bolivia, Argentina and Peru including the living type collection (*Weigend et al. 97/450* – F, M, USM) of *X.klaprothioides* at Bonn University Botanic Gardens. Likewise, we studied previous *X.klaprothioides* collections, now deposited in B, F, HUT, M and USM (all made by MW, TH and their field companions since 1997). The listed herbarium acronyms follow the Index Herbariorum abbreviations (Thiers 2021). The conservation status was assessed using GeoCAT (Bachman et al. 2011). The photographic plates showing different parts of living plants, are adapted from the LCDP-technique developed by Pupulin and Bogarín (2004). The colored SEM images for figure 5 were prepared by H.J. Ensikat (Bonn) in the course of studies on the biomineralization of trichomes in Loasaceae. For methodological details see Ensikat et al. (2017). The distribution map was prepared using ArcGIS.

### ﻿Taxonomic treatment

#### ﻿ Xylopodia

Taxon classificationPlantaeCornalesLoasaceae

Weigend, Taxon 55: 467. 2006.

326F4FC1-0638-529C-AA0B-698450576ADE

##### Type species.

*Xylopodiaklaprothioides* Weigend.

##### Description.

Shrubs with erect branches from horizontal xylopodia; aerial vegetative organs densely hairy, with scabrid and glochidiate trichomes. Leaves simple, opposite, estipulate, petiolate; lamina with 1–3 lobes on each side, margin serrate. Inflorescences terminal dichasia, each flower with 2 bracts. Flowers tetramerous, deflexed. Sepals triangular. Petals cymbiform, greenish-white to green, aestivation valvate. Nectar scales 4, antesepalous, white and green or green and yellow, formed by 4–6 fused staminodes. Free staminodes 4–5, opposite and internal relative to the nectar scale. Fertile stamens arranged in 4 antepetalous groups. Ovary semisuperior with 4 placentae. Fruits semisuperior capsules, subglobose, straight, opening with 4 apical valves. Seeds narrowly ovoidal, testa papillose-reticulate. 2N = 24.

##### Etymology.

This name makes reference to the well-developed xylopodia (ligneous rhizomes) found in the type species of the genus.

##### Distribution.

So far, *Xylopodia* is known in a few widely separated, disjunct localities in strongly seasonal habitats of the tropical Andes in NW Peru, NW and S Bolivia and NW Argentina, from low (850–900 m) to moderate (2,900 m) elevations.

##### Identification remarks.

Plants of this genus can be recognized from all other Loasaceae by the diagnostic combination of the presence of underground xylopodia, opposite, lobed leaves, tetramerous flowers, petals with longitudinal lamellae and well developed nectar scales.

#### ﻿ Xylopodia klaprothioides

Taxon classificationPlantaeCornalesLoasaceae

Weigend, Taxon 55: 467. 2006.

D1AFEF9B-1D85-5DA9-8209-E250741FF5A9

Figs 2 , 5

##### Type.

Peru. Cajamarca: Prov. Contumazá: Road from Contumazá to Chilete, first roadbend after highest point pass, 2900 m, (07°19'48"S, 078°48'38.5"W) 5. Feb. - 2. Abr. 1997, *M. Weigend*, *N. Dostert & K. Drießle 97/450* (***holotype***: M! mounted on two sheets, barcodes: M-0274954 & M-0274955; ***isotypes***: F!, USM!).

##### Description.

Shrub with erect or leaning shoots up to ca. 300 cm tall from a horizontal xylopodium (= ligneous rhizome), up to 30 × 5 cm, with several aerial branches arising from it; aerial vegetative organs densely hairy, with white scabrid and glochidiate trichomes, ca. 1 mm long. Leaves opposite, 8–20 × 65–15 cm, lamina with 1–3 lobes on each side, margin serrate, apices acute to rounded, base cuneate, petiole 1–3 cm long. Inflorescences terminal dichasia; peduncle ca. 7–20 cm long; basal bracts 1.5 × 0.5 cm, 3-lobed, margin denticulate, base cuneate; distal bracts 0.5 × 0.1 cm, lanceolate, subentire. Flowers tetramerous, pedicels 2–4 mm long, deflexed. Sepals broadly triangular, ca. 3 × 2 mm, margins entire, erect in bud, spreading during anthesis, and pubescent on both sides, pubescence similar to that of vegetative organs. Petals 10–15 × 3–5 mm, pale to deep green, unguiculate, with a short claw (< 1/4 of petal length), cucullate in the distal half, with two membranous longitudinal lamellae with densely ciliate margin, aestivation valvate, margin with a tooth on each side near the base where the limb starts, with scabrid-glochidiate trichomes abaxially, adaxially glabrous. Nectar scales 4, antesepalous, 3.5–4 × 2.5–3 mm, cucullate, formed by 5–6 fused staminodes, mostly green, pubescent in lower half, virtually uniform in color, apex yellowish green, slightly recurved. Free staminodes 4–5 per scale, opposite and internal to it, monomorphic, lower halves densely pubescent, green, ca. 4 mm long, with an inconspicuous, pubescent, knee-like filament flange below the middle, apex filiform, twisted randomly or reflexed. Fertile stamens arranged in 4 antepetalous groups of ca. 7–15 per petal, free, filament ca. 6–8 mm long, anthers white, with two ovate thecae. Ovary half-superior, broadly ovate to conical, placentae 4; style to 7 mm long, lower half pubescent, stigma with four ribs tapering towards the apex. Fruits subglobose capsules, with a conical apex, opening with 4 apical valves. Seeds numerous, narrowly ovoidal, testa papillose-reticulate up to ca. 2 × 0.4 mm.

**Figure 3.**
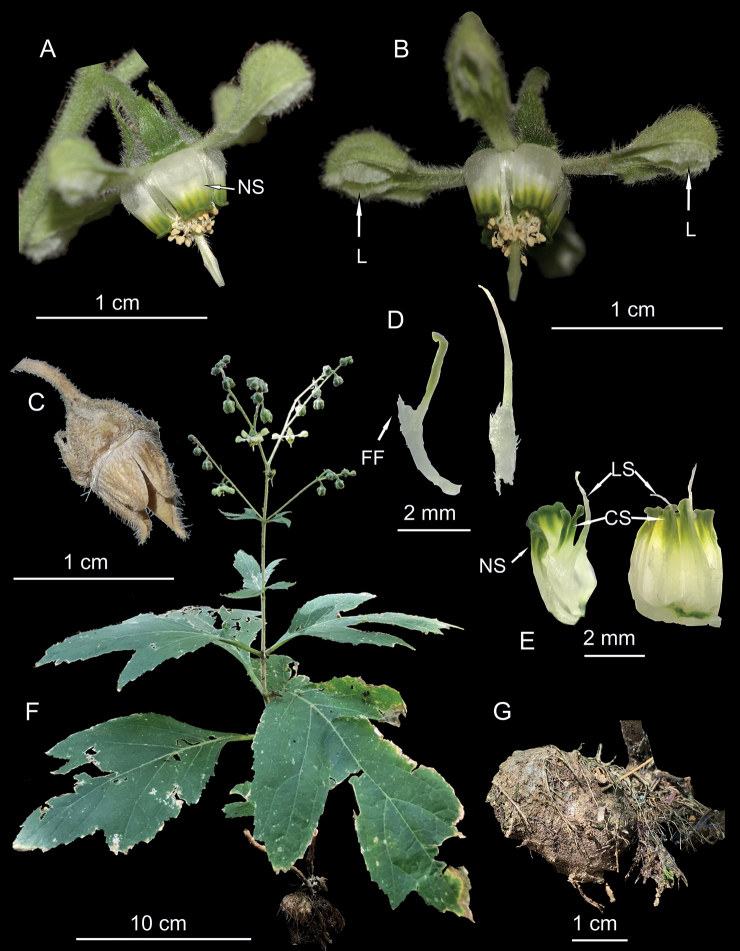
*Xylopodialaurensis*. **A** flower, lateral view, showing the nectar scales (NS) **B** flower, showing the lamellae (L) **C** mature fruit, showing the four apical valves **D** details of the central staminode (left), showing the well-developed filament flange (FF), lateral staminode (right) **E** staminodes within the nectar scale, showing the lateral staminodes (LS) and the central ones (CS) **F** plant **G** xylopodium. All from *Martín 2887*, except C (*Catari 2510*).

##### Etymology.

The species name refers to the clear affinity of this taxon to the genus *Klaprothia* Kunth.

##### Distribution.

*Xylopodiaklaprothioides* is restricted to a small area of the Pacific slope of the Cordillera Occidental in Andean NW Peru. It is a very narrowly endemic species, known from only two localities some 8 km apart (in a direct line) in Dpto. Cajamarca Prov. Contumazá, at elevations from ca. 2,500–3,000 m (Fig. 1).

##### Ecology.

**Figure 4.**
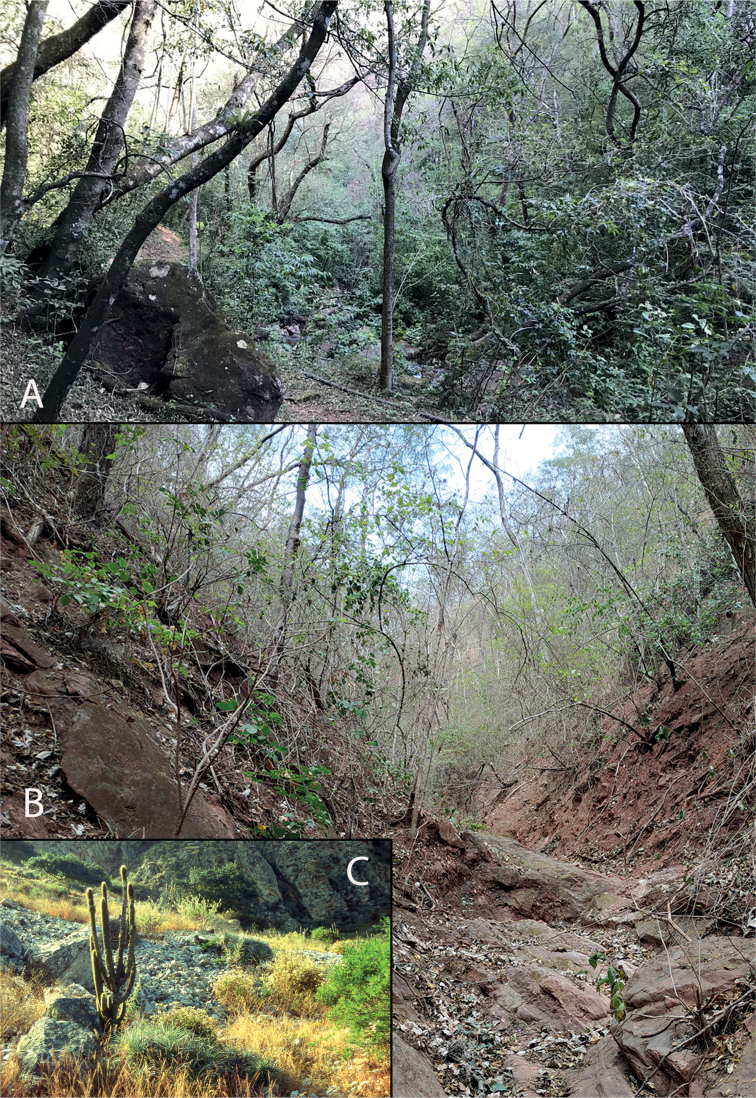
Habitats of *Xylopodia*. **A** deciduous forest near Jujuy, Argentina, habitat of *X.laurensis***B***X.laurensis* habitat in a dry creek near Chuquisaca, Bolivia **C** rocky slopes with xerophilous vegetation near the town of Contumazá, Peru, habitat of narrowly endemic *X.klaprothioides*.

*Xylopodiaklaprothioides* occurs in seasonally dry Andean scrub habitats, mostly around hedges and gullies, often on rocky soil dominated by xeric adapted shrubs, small trees and other vegetation including *Cylindropuntia* (Engelm.) F.M. Knuth and terrestrial bromeliads (Fig. 4C). Near the town of Contumazá, it grows sympatrically with another shrubby Loasaceae, *Nasamacrothyrsa* (Urb. & Gilg) Weigend. These localities are surrounded by a mosaic of fields, fallows and small patches of seasonally dry montane forest according to the Ministerio del Ambiente del Perú (2015, 2018). These habitats often show signs of human alteration. Rodríguez-Rodríguez and Weigend (2006) mention that this species is found in the Mesoandina Ecological Region. *Xylopodiaklaprothioides* is deciduous and the plants shed their leaves at the beginning of flowering in May/June.

##### Phenology.

Flowering and fruiting of this species have been recorded at the very end of the rainy season and the start of the dry season in May and June; thus most plants are leafless during the peak of the flowering period.

##### Additional specimens examined.

Perú: Dpto. Cajamarca, Prov. Contumazá, Road from Contumazá to Chilete. First road bend after highest point of pass, 2900 m, (07°19'48"S, 078°48'38.5"W) 17. Jun. 1998, *M. Weigend et al. 98/536* (F, HUT, M, USM); same locality, 09. May 2003, *M. Weigend et al. 7601* (B, F, HUT, M, USM); Dpto Cajamarca, Prov. Contumazá, road Contumazá to Cascas, after tunnel, ca. 2400–2600 m, (07°24'14"S, 078°47'53"W) 10. May 2003, *M. Weigend et al. 7617* (B, F, HUT, M, USM).

##### Notes.

See *Xylopodialaurensis*.

##### Conservation status.

*Xylopodiaklaprothioides* seems to be restricted to only a few populations near Contumazá in northern Peru and has been considered as globally endangered (Rodríguez and Weigend 2006). Using the data from the only two known populations, the estimated EOO (Extent Of Occurrence) of the species is just 0.081 km^2^, resulting in a conservation status assessment of “critically endangered” (CR) according to the IUCN categories and criteria (2012) and guidelines (2019). The AOO (Area Of Occupancy) for the species is also extremely small (8 km^2^ when applying a reasonable grid cell size of 2 × 2 km in GeoCat) and also assigns *X.klaprothioides* a “critically endangered” (CR) status according to the IUCN. We thus would update the threatened assessment of this species to CR under the IUCN categories and criteria B2ab(iii); C2a(i), The criterion B2 was selected because its AOO is < 10 km^2^ (8 km^2^). The criterion “a” was selected because it presents a very fragmented distribution. The criterion “b(iii)” was selected because there is a projected decline in the area, extent and quality of habitat. The region around Contumazá represents a mosaic of different habitats including small patches of natural habitat surrounded by agricultural land. The criterion C2a(i) was selected because we observed fewer than 50 individuals per population in the two known localities.

#### ﻿ Xylopodia laurensis

Taxon classificationPlantaeCornalesLoasaceae

C.M. Martín & C.A. Zanotti sp. nov.

8B6F06DC-FFEA-559B-AF86-261F793A9D6F

urn:lsid:ipni.org:names:77297017-1

Fig. 3

##### Diagnosis.

*Xylopodialaurensis* is closely related to *Xylopodiaklaprothioides* Weigend, but the former is readily distinguishable by the sepals reflexed in anthesis, petals greenish white with a long claw (ca. 1/3 of petal length); back of nectar scale white, apically striped green and yellowish; free staminodes are dimorphic, ligulate, the central pair with a conspicuous, papillose, scale-like filament flange below the middle.

##### Type.

Argentina: Prov. Jujuy, Dpto. San Pedro, San Juan de Dios, Estancia Las Lauras, camino al “Chorro”, a tres metros del arroyo Chico, 24°33'49.8"S, 64°39'05.8"W, 925 m, 22 Aug 2021, *C. M. Martín 2887* (holotype SI)

##### Description.

Shrub with erect shoots up to 130 cm tall from a short horizontal xylopodium (= ligneous rhizome), ca. 5 × 3 cm, sometimes divided into 3–4 branches distally; aerial vegetative organs densely hairy, with white scabrid and glochidiate trichomes, ca. 0.5 mm long. Leaves opposite, 14–22 × 13–9 cm, lamina with 1–2 lobes in each side, margin serrate, apices acute to rounded, base cuneate, petiole 1.5–3 cm long. Inflorescences terminal dichasia; peduncle ca. 6 cm long; basal bracts 3 × 2 cm, 3-lobed, margin denticulate, base cuneate; distal bracts 0.8–1.8 × 0.3–0.5 cm, lanceolate, margin entire. Flowers tetramerous, pedicels 4–6 mm long, deflexed. Sepals broadly triangular, ca. 5 × 3 mm, margins entire, erect in bud, reflexed during anthesis, and pubescent on both sides, pubescence similar to that of vegetative organ. Petals 7 ×3 mm, greenish white, unguiculate, with a long claw (ca. 1/3 of petal length), cucullate in the distal half, with two membranous longitudinal lamellae with densely ciliate margin, aestivation valvate, margin with a tooth on each side near the middle, where the limb starts, dorsal veins three, with scabrid-glochidiate trichomes abaxially, mostly glabrous adaxially. Nectar scales 4, antesepalous, 4 × 3 mm, cucullate, formed apparently by 6 fused staminodes, mostly white, pubescent in lower half, striped green and yellowish in distal third, apex green, slightly recurved. Free staminodes 4 per scale, opposite and internal to it, dimorphic, their lower halves partly fused, densely pubescent, with laciniate margins, central staminodes green, 4 mm long, with a conspicuous, papillose, scale-like filament flange below the middle, apex reflexed, spathulate the lateral ones yellowish-white, 5.3 mm long, with a filiform apex. Fertile stamens arranged in 4 antepetalous groups of ca. 7 per petal, free, filament ca. 6 mm long, base pubescent, anthers white, with two obovate thecae. Ovary half-superior, broadly ovate to conical; style to 5 mm long, lower half pubescent, stigma with four ribs tapering towards the apex. Fruits subglobose capsules, with a conical, slightly asymmetrical apex, opening with 4 apical valves.

##### Etymology.

The name of the species refers to the locality of the type collection in Argentina (Estancia Las Lauras, Jujuy, Argentina).

##### Distribution.

*Xylopodialaurensis* grows on the eastern slope of the Andes from Bolivia (Dptos. La Paz and Chuquisaca) to northern Argentina (Prov. Jujuy) at elevations from 850–900 m asl (Jujuy and Chuquisaca) to 2360 m asl (La Paz) (Fig. 1). The known distribution thereby covers a latitudinal range of ca. 1000 km in a straight line between the northernmost locality in Dpto. La Paz and the southernmost population from Prov. Jujuy. The distributional range is even larger (~1300 km) when following the curve of the eastern slope of the Andes along a suitable elevation with appropriate climatic conditions and respective available habitats. *X.laurensis* can hence be considered a rather widespread taxon, albeit with very specific saxicolous habitat requirements, and this contrasts markedly with the very narrow endemism of its sister taxon, *X.klaprothioides*. Narrow endemism as found in *X.klaprothioides* is a common pattern reported for many plant groups whose elements reach into the Amotape-Huancabamba zone (e.g. Gentianaceae – Struwe et al. 2009; Boraginaceae – Weigend et al. 2010; Grossulariaceae, Urticaceae – Mutke et al. 2014; Solanaceae – Deanna et al. 2018; Arecaceae – Escobar et al. 2018; Lentibulariaceae – Casper et al. 2020; Henning et al. 2021) and particularly common in the Loasaceae (Henning and Weigend 2009; Henning et al. 2011, 2019; Mutke et al. 2014).

##### Ecology.

*Xylopodialaurensis* occurs in the understory of seasonally dry (scrub) forest in shallow rocky soil and in soil pockets on rock-outcrops across its range (Fig. 4). In Argentina, it grows in xerophilous and deciduous forests (Fig. 4A) at 925 m a. s. l. corresponding to the Chaco Occidental phytogeographic district sensu Cabrera 1994. Typical components are *Schinopsislorentzii* (Griseb.) Engl., *Libidibiaparaguariensis* (D. Parodi) G.P. Lewis, *Handroanthusimpetiginosus* (Mart. ex DC.) Mattos, *Ceibachodatii* (Hassl.) Ravenna, *Aspidospermaquebracho-blanco* Schltdl. and *Athyanaweinmanniifolia* (Griseb.) Radlk. In Chuquisaca, *Xylopodialaurensis* forms small colonies on rocky outcrops and the margins of temporary streams (torrenteras, Fig. 4B) in the Boliviano-Tucumano Seasonal Dry Forests (Pilcomayo-Alto Parapeti Sector of the Boliviano-Tucumano Biogeographic Province sensu Navarro and Maldonado 2002 and Navarro and Ferreira 2009). The most common species here are *Schinopsislorentzii*, *Anadenantheracolubrina* (Vell.) Brenan, *Ceibachodatii*, *Saccelliumlanceolatum* Bonpl., *Piptadeniaboliviana* Benth., *Ruprechtiaapetala* Wedd. and *Libidibiaparaguariensis*. These rock outcrop habitats are poorly studied in Bolivia and several new species from other plant groups still await description (JC unpublished data). In La Paz, the new species is found in the Yungueño Montane semi-deciduous forest. This area has been more profoundly influenced by human activities. The original forests have been replaced by scrub and secondary forests (these correspond to the Cuenca Alta del Beni Sector of the Yungueña Peruviana-Boliviana Biogeographic Province sensu Navarro and Maldonado 2002 and Navarro and Ferreira 2009).

##### Phenology.

The flowering and fruiting of this species have been recorded in the dry season, from late August to mid-September in Jujuy and from April to August in Chuquisaca. In Chuquisaca, plants lose their foliage by August and produce new leaves when the rainy season starts in October to November.

##### Additional specimens examined.

Bolivia: Dpto. Chuquisaca. Prov. Luis Calvo, Municipio Villa Vaca Guzmán, A 5.5 km al Norte de la Comunidad Ivoca y 3 km al Este de la Quebrada Angoba. Estancia del Sr. Jhony Labras. 850 m, 11. Sep. 2019, *J.C. Catari & Z. Pérez 2501* (USZ); same locality, 17. Oct. 2021, *J.C. Catari 2510* (USZ). Photographs examined: Bolivia: Dpto. La Paz, Prov. Larecaja, Municipio Sorata, Cueva de San Pedro, s.d., autor: A.F. Fuentes (available in http://legacy.tropicos.org/Image/100494035)

##### Notes.

*Xylopodialaurensis* is a close ally of *Xylopodiaklaprothioides*. Vegetatively, it mainly differs in the much smaller woody rhizome, which easily reaches 30 cm and more in length in mature *X.klaprothioides*. Aerial stem and leaf morphology appear to be similar, although the leaves of *X.klaprothioides* are usually smaller and not as deeply lobed (Figs 2F, 3F). The major differences, however, are found in the details of floral morphology, where *X.laurensis* differs in its reflexed (versus spreading) sepals during anthesis (Figs 2A–B, 3A–B), floral scale coloration (white and yellowish-green pattern apically versus uniformly green with yellowish scale neck, Figs 2A–B, D, 3A–B, E) and the morphology of the staminodes (Figs 2E, 3D). The central staminodes are particularly distinctive and their enlarged filament flanges resemble those of *Kisseniacapensis* R.Br. ex Harv. (Urban and Gilg 1900).

**Figure 5.**
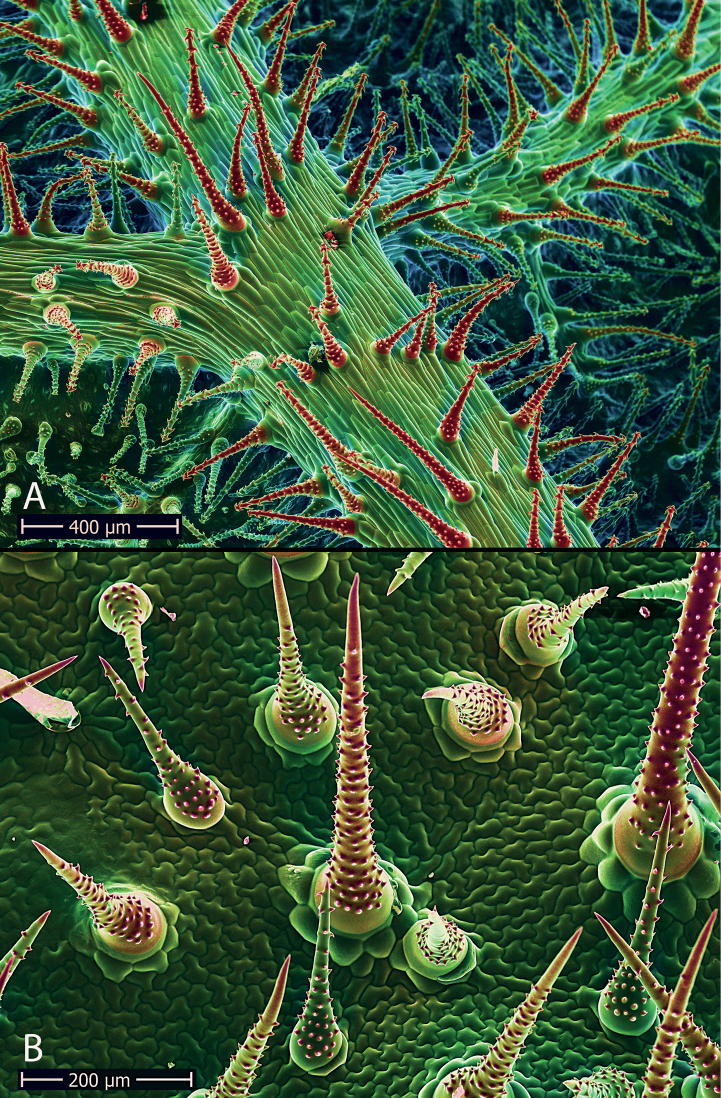
False-color BSE-image of the trichome cover of the leaves of *X.klaprothioides*. Mineralized areas are highlighted in red, non-mineralized areas in green **A** abaxial surface densely covered with unicellular, glochidiate and a few scabrid trichomes **B** adaxial surface densely set with scabrid hairs. (Credit: H.J. Ensikat, Bonn).

There is a disjunction of ca. 1500 km between the populations of *X.klaprothioides* in northern Peru and *X.laurensis* in Bolivia (Fig. 1). The recent discovery of this genus demonstrates that it is very difficult to locate in the field – mostly because it has few if any leaves when in flower, and the green flowers are hardly differentiated from the surrounding vegetation, rendering visual recognition of even flowering plants a genuine challenge. If found when sterile, it could be confused with some other oppositely leaved subshrubs or shrubs (high magnification lenses are necessary to see the distinctive scabrid-glochidiate trichomes, Fig. 5). Likewise, the species, even if widely distributed, could be very localized in distribution. One of us (JC) explored an area of ca. 10 ha of suitable habitat near the collection locality in Chuquisaca and was able to locate only two small colonies distributed in an area of < 1000 m^2^. It is therefore conceivable that *Xylopodia* is also present in similar vegetation in the intervening area between the known ranges of these two species, for example in the poorly explored Río Mantaro and Río Apurímac systems in Peru. Future explorations may turn up additional localities for this species. However, as it now stands, it appears to represent a highly unusual disjunction shown by few other plant taxa restricted to the seasonally dry forests of northwestern Peru and the eastern Andean slope in Bolivia and Argentina, corresponding to what Prado and Gibbs (1993) consider as the Pleistocene arc of tropical seasonal dry forests.

##### Preliminary conservation status.

As mentioned above, the actual abundance of *Xylopodia* is very difficult to assess, both globally and locally. Both species share a very nondescript appearance, even during flowering and especially after the shedding of leaves during the dry season. Hence, there are only very few collections. However, unlike the type species, *X.laurensis* is evidently more widespread and likely present in suitable habitats in between the known populations (Fig. 1). The estimated EOO (Extent Of Occurrence) is > 72.000 km^2^, resulting in a conservation status assessment of “least concern” (LC) according to the IUCN categories and criteria (2012) and guidelines (2019). Conversely, given the fact that all collections and observations made so far only report very small populations or single individuals, the AOO (Area Of Occupancy) for the species is extremely small (<0.03 km^2^ when applying the smallest possible grid cell of 100 × 100 m in GeoCat) presumably reflecting the narrowness of the ecological niche of *Xylopodia*. The latter value would consider *X.laurensis* as “critically endangered” (CR) according to the IUCN. These contradictory results show how difficult even a tentative assessment of a species’ conservation status can be, if the data are too limited due to either collection gaps in certain regions or taxonomic groups or real rarity of the taxon in question. At the moment, we cannot give a satisfactory answer on the threat status of *X.laurensis*. It might be rare and under immediate threat or maybe it has been just under collected. For the time being its conservation status must hence be categorized as “data deficient” (DD).

### ﻿Key to the Species of *Xylopodia*

**Table:** 

1	Sepals reflexed at anthesis, petals greenish white, claw long (ca. 1/3 of length of petals); back of nectar scale white, apically striped green and yellowish, apex green; free staminodes dimorphic, with their lower halves partly fused, the central pair abruptly expanded basally into unusually large, scale-like,abaxial filament flanges ca. ½ from base	***X.laurensis***
–	Sepals spreading at anthesis, petals pale to deep green, claw short (< 1/4 of length of petals); back of nectar scale uniformly green, apex yellowish-green; free staminodes monomorphic, free, with small and inconspicuous “knee shaped” abaxial filament flanges ca. ¼ from base	***X.klaprothioides***

## Supplementary Material

XML Treatment for Xylopodia

XML Treatment for Xylopodia klaprothioides

XML Treatment for Xylopodia laurensis
